# Supplementary figures and images for: Dendritic Voltage Recordings Explain Paradoxical Synaptic Plasticity: A Modeling Study
Source: Front Synaptic Neurosci. 2020 Nov 2;12:585539. doi: 10.3389/fnsyn.2020.585539 (PMC7670913; doi:10.3389/fnsyn.2020.585539)

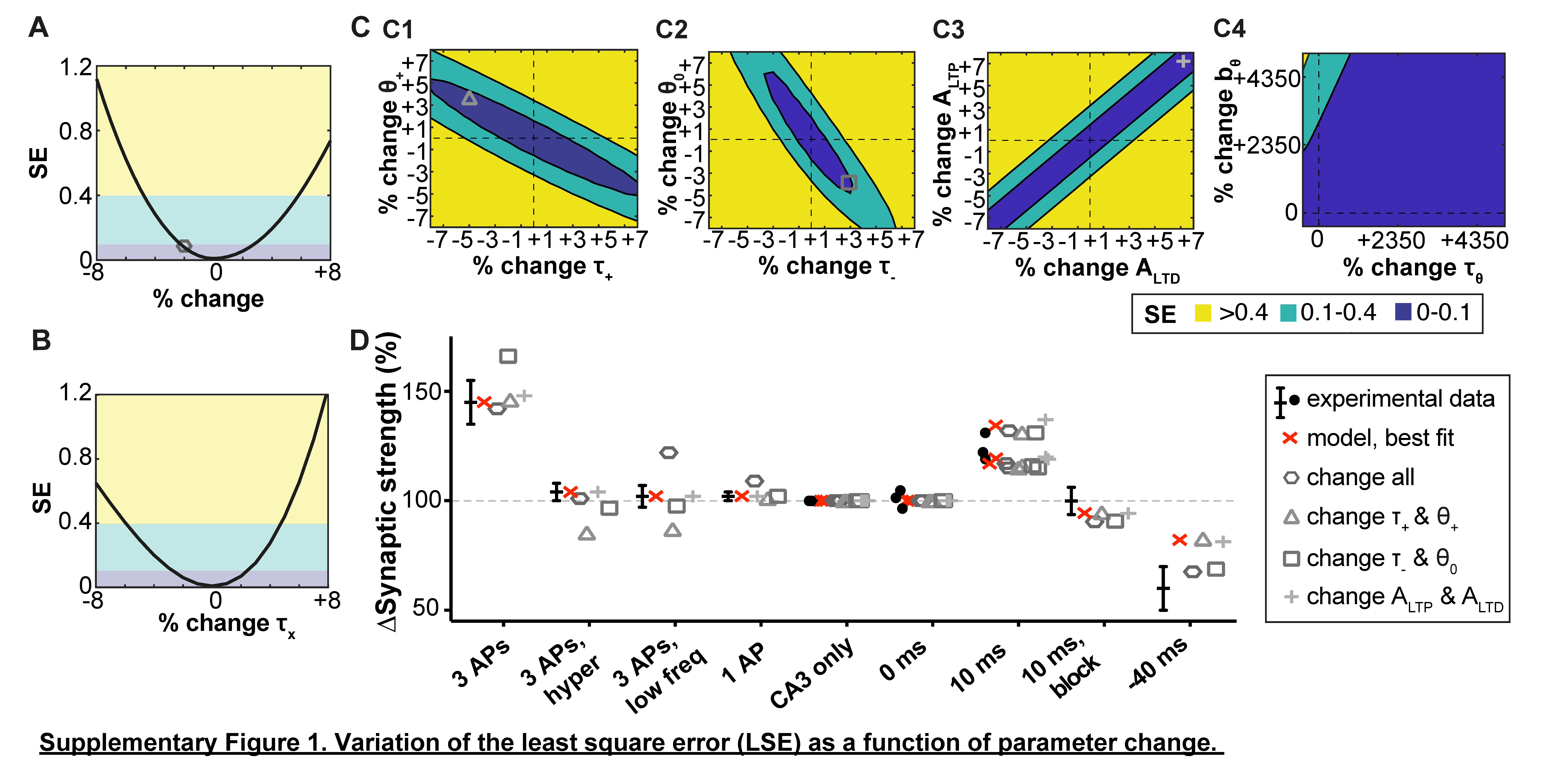

Supplement: Supplementary Figure 1 — Variation of the squared error (SE) as a function of parameter change. (A) SE (vertical axis) when all the parameters (see Table 1) were increased or decreased (horizontal axis) by a fixed percentage. (B) Only the parameter τx is changed (horizontal axis) by a fixed percentage. (C) Filled contour plot of the SE while 2 parameters are increased or decreased by a given percentage: τ+ & θ+ (C1), τ– & θ0 (C2), ALTP & ALTD (C3) bθ & τθ (C4). (D) Plasticity value in 9 different conditions (see Figure 2). Black circles and error bars represent experimental data. Red crosses represent simulations using the parameters obtained with the best fit. Gray symbols in D represent simulations using the parameters obtained with the best fit except a few which were changed by a certain percentage or when all parameters were changed by a fixed percentage (compare symbols in A,C1–C3): hexagon in (A) (−2%), upwards-pointing triangle in (C1) (−5 and +4%), rectangle in (C2) (+3 and −4%), cross in (C3) (+7 and +7%). [file Image_1.tiff]
